# Severe acute pancreatitis: surgical indications and treatment

**DOI:** 10.1007/s00423-020-01944-6

**Published:** 2020-09-10

**Authors:** Max Heckler, Thilo Hackert, Kai Hu, Cristopher M. Halloran, Markus W. Büchler, John P. Neoptolemos

**Affiliations:** 1 Department of General, Visceral and Transplantation Surgery, University of Heidelberg, Im Neuenheimer Feld 110, 69120 Heidelberg, Baden-Württemberg Germany; 2 Department of Molecular and Clinical Cancer Medicine, University of Liverpool, Liverpool, UK

**Keywords:** Pancreatic necrosis, Infection, Minimally invasive surgery, Necrosectomy, Endoscopic, Percutaneous

## Abstract

**Background:**

Acute pancreatitis (AP) is defined as an acute inflammatory attack of the pancreas of sudden onset. Around 25% of patients have either moderately severe or severe disease with a mortality rate of 15–20%.

**Purpose:**

The aim of this article was to summarize the advances being made in the understanding of this disease and the important role of surgery.

**Results and conclusions:**

An accurate diagnosis should be made a soon as possible, initiating resuscitation with large volume intravenous fluids and oxygen by mask. Predicted severe disease will require intensive monitoring. Most deaths within the first week are due to multi-organ failure; thus, these patients will require intensive therapy unit management. During the second phase of the disease, death is due to local complications arising from the pancreatic inflammation, requiring accurate identification to determine the correct form of treatment. Acute peripancreatic fluid collections arise < 4 weeks after onset of interstitial edematous pancreatitis, not requiring any treatment. Most pancreatic pseudocysts arise > 4 weeks and largely resolve on conservative management. Necrotizing pancreatitis causing acute necrotic collections and later walled-off necrosis will require treatment if symptomatic or infected. Initial endoscopic transgastric or percutaneous drainage will resolve less serious collections but necrosectomy using minimally invasive approaches will be needed for more serious collections. To prevent recurrent attacks of AP, causative factors need to be removed where possible such as cholecystectomy and cessation of alcohol. Future progress requires improved management of multi-organ failure and more effective minimally invasive techniques for the removal of necrosis.

## Introduction

### Etiology, incidence, financial aspects

Acute pancreatitis (AP) is defined as an acute inflammatory attack of the pancreas with a sudden onset of symptoms, which, in the absence of post necrotic damage to the gland, results in complete resolution of histology, physiology, and symptoms and provided the initiating cause is removed there will be no further attacks. The commonest causes for AP are gallstones (40–65%) and alcohol (25–40%), and the remainder (10–30%) are due to a variety of causes including autoimmune and genetic risk factors (Table 1) [1, 2]. Irrespective of etiology, the trigger factors cause supraphysiological intracellular signaling resulting in trypsin activation within the zymogen granules [3–5]. The resultant acinar cell death causes a localized and systemic inflammatory response. Initially, the most prominent features are distant organ dysfunction notably the lungs and kidneys, which in most cases is of short duration (< 48 h) [6, 7].

**Table 1:** Causes and risk factors for acute pancreatitis

Causes	Sub-types	Comments
Gallstones		40–65%
Toxic-metabolic		
Alcohol		25–40% Risk factor
Tobacco smoking		Risk factor
Hypercalcemia	Hyperparathyroidism	
Hypertriglyceridemia		Not hyperlipidemia Caution: alcohol pancreatitis can induce hypertriglyceridemia
Chronic kidney disease		
Medications	Acne treatments—tetracycline, isotretinoin, Roaccutane, cannabis, carbimazole, furosemide, isoniazid, metronidazole, simvastatin	Definite causality; others
Chemotherapy		
Radiation		
Porphyria	Acute intermittent porphyria Erythropoietic protoporphyria	
Toxins	Scorpion sting—Trinidad thick-tailed scorpion (Tityus trinitatis). Snake bites—adder (*Vipera berus*), common krait (Bungarus caeruleus), viper (*Cerastes cerastes*). Hymenoptera—hornets.	
Chemical	Penetrating duodenal or gastric peptic ulcers.	
Idiopathic	Early onset Late onset	10–30% Not gene-related
Obstructive	Ampullary stenosis/tumors, main duct strictures; pancreatic tumors, IPMN, lymphoma, pancreas divisum with duct narrowing, annular pancreas, pancreatobiliary maljunction, choledochocele, intraluminal duodenal diverticulum	
Trauma	Blunt abdominal. Iatrogenic surgical—renal surgery, organ transplantation, partial pancreatectomy. Iatrogenic endoscopic—ERCP, EUS biopsy	
Genetic		
Hereditary pancreatitis	PRSS1 gene mutations CPA1 gene mutations	Autosomal dominant Highly penetrant
Cystic fibrosis	CFTR gene mutations	Autosomal recessive
Genetic risk factors	SPINK1, CFTR, CTRC, CEL, CPA1, and PRSS1 gene variants and/or mutations	Increase risk in alcohol and idiopathic acute pancreatitis
Autoimmune	Autoimmune pancreatitis—predominantly type II. Syndromic—SLE, vasculitis	
Infection	Viruses—Coxsackie B, CMV, Covid19, EBV, Hep B, HIV, HSV, mumps, varicella-zoster. Bacteria—legionella, leptospira, mycoplasma, salmonella. Fungi—aspergillus. Parasites—ascaris, cryptosporidium, toxoplasma, clonorchiasis.	
Ischemia and embolism	Cardiac surgery, abdominal aorta dissection	

The incidence of AP is rising globally with an estimate of 34 cases (95% confidence interval (Cl) 23–49) per 10^5^ general population per year [8]. In Europe, the incidence of AP ranged from 4.6 to 100 per 10^5^ population and was the highest in eastern and northern Europe [9]. In the USA, there were approximately 275,000 hospitalizations in 2009, almost doubling from that in 1988 [2]. Longitudinal data from Japan demonstrate a threefold increase from 1998 to 2011, with a prevalence of 49.4 per 10^5^ [10]. Lifestyle factors probably account for the rising incidence, which can be attributed to diet and gallstones, alcohol and smoking consumption, diabetes mellitus, and obesity [2]. The overall financial burden of AP on public health systems across the world is considerable. A USA study from 2007 estimated $2 billion for hospitalization for AP—around $10,000 per admission [11]. Timely identification of patients at risk for developing severe disease and a state-of-the-art treatment of these patients are multidisciplinary challenges.

### Classification and severity

**Fig. 1:**
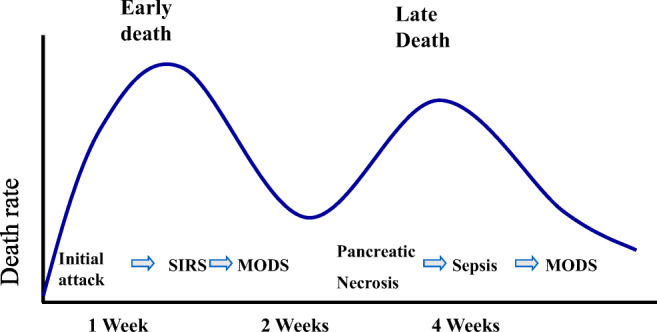
Distribution of deaths in patients with severe pancreatitis. Most deaths occur in the first week or so from multi-organ dysfunction syndrome (MODS) consequent to an excessive systemic inflammatory response syndrome (SIRS). In the second phase, deaths tend to occur from pancreatic necrosis and are associated with sepsis, leading to secondary MODS

Around 75% of cases of AP have a mild clinical course and are self-limiting, usually only requiring intravenous fluids with oxygen support by mask [7]. The remaining patients are classified as having either moderately severe or severe disease with a mortality rate of 15–20% [12–15]. Persistent organ failure beyond 48 h is the major cause of morbidity occurring in around half of the patients with pancreatic necrosis and in up to two-thirds of those with superimposed infection [12–15]. Pancreatic necrosis develops in approximately 20% of patients, with infection of the pancreatic necrosis occurring in 30–70% of patients resulting in a mortality rate of 20–30% [13–16]. Fungal infection may occur secondarily to bacterial infection and is associated with high mortality in primary and secondary infected pancreatic necrosis, requiring aggressive systemic anti-fungal therapy [17]. There are two phases of mortality, the major cause of death in the first week being continuing multiple organ failure, while most deaths in the subsequent period are due to local pancreatic necrosis (Fig. 1). In a systematic review and meta-analysis totaling 6970 patients, the mortality rate in patients with infected necrosis and organ failure was 35.2%, compared with 19.8% for sterile necrosis with organ failure and 1.4% for infected necrosis without organ failure [18].

The revised Atlanta classification published in 2012 provides a well-established framework for the stratification of AP patients with precise definitions of complications and severity shown in Table 2 [12]. Correct identification of the nature of the local complication is important for clinical decision-making. The Determinant-Based Classification provides an additional Critical Severity Grade defined as a combination of both infected pancreatic necrosis and persistent organ failure, but is not as widely used as the revised Atlanta [19]. The recommendations of the IAP/APA evidence-based guidelines for the management of acute pancreatitis incorporate the 2012 revised Atlanta classification and are summarized in Table 3 [20].

**Table 2:** Definitions of the 2012 Atlanta classification revision

Definition	Contrast-enhanced computed tomography criteria
Interstitial edematous pancreatitis Acute inflammation of the pancreatic parenchyma and peripancreatic tissues, but without recognizable tissue necrosis.	Pancreatic parenchyma enhancement by intravenous contrast agent.
Necrotizing pancreatitis Inflammation associated with pancreatic parenchymal necrosis and/or peripancreatic necrosis.	Lack of pancreatic parenchymal enhancement by intravenous contrast agent and/or peripancreatic necrosis.
Acute peripancreatic fluid collection (APFC) Peripancreatic fluid associated with interstitial edematous pancreatitis with no associated peripancreatic necrosis—< 4 weeks after onset of interstitial edematous pancreatitis and without a pseudocyst.	Interstitial edematous pancreatitis: homogeneous collection with fluid density, confined by normal peripancreatic fascial planes, without wall encapsulation, and adjacent to the pancreas without intra-pancreatic extension.
Pancreatic pseudocyst An encapsulated collection of fluid with a well-defined inflammatory wall usually outside the pancreas with minimal or no necrosis—> 4 weeks after onset of interstitial edematous pancreatitis.	Well circumscribed, usually round or oval, well-defined wall that is, completely encapsulated, homogeneous fluid density, no non-liquid component.
Acute necrotic collection (ANC) A collection containing variable amounts of both fluid and necrosis associated with necrotizing pancreatitis; the necrosis can involve the pancreatic parenchyma and/or the peripancreatic tissues.	Acute necrotizing pancreatitis: Heterogeneous and non-liquid density of varying degrees in different locations (some appear homogeneous early in their course). No definable wall encapsulating the collection intra-pancreatic and/or extra-pancreatic.
Walled-off necrosis (WON) A mature, encapsulated collection of pancreatic and/or peripancreatic necrosis with a well-defined inflammatory wall, > 4 weeks after onset of necrotizing pancreatitis.	Heterogeneous with liquid and non-liquid density with varying degrees of loculations (may appear homogeneous initially), well-defined completely encapsulated wall, intra-pancreatic, and/or extra-pancreatic.
Severity of acute pancreatitis	Severity criteria
Mild acute pancreatitis	▸ No organ failure ▸ No local or systemic complications
Moderately severe acute pancreatitis	▸*Transient organ failure* that resolves < 48 h and/or ▸ Local or systemic complications without persistent organ failure
Severe acute pancreatitis	▸ *Persistent organ failure* that persists > 48 h – Single organ failure – Multiple organ failure

**Table 3:** IAP/APA evidence-based guidelines for the management of acute pancreatitis

	Domain of guidelines	Level of evidence; level of agreement
A	Diagnosis and etiology of AP	
1	2 out of 3 criteria (upper abdominal pain, imaging (CT, EUS, US), elevation of serum amylase/lipase over threefold) are needed for diagnosis.	1B; strong *t*
2	Etiology should be determined on admission (history, imaging, examination, laboratory tests).	1B; strong agreement
3	For idiopathic AP, EUS should be performed as a next step for microlithiasis detection. If negative, MRCP is recommended. If inconclusive, genetic counseling can be evaluated.	2C; weak
B	Prognostication/prediction of severity	
4	SIRS is advised to predict severe AP at admission and persistent SIRS at 48 h.	2B; weak
5	A 3-dimension approach (host risk factors, clinical risk stratification, response to initial therapy) is advised to predict outcome of AP.	2B; strong
C	Imaging	
6	Indications for initial CT include diagnostic uncertainty, confirmation of severity, failure to respond to conservative treatment. Optimal timing is 72–96 h after symptom onset.	1C; strong
7	Indications for follow-up CT are lack of clinical improvement, deterioration, planned invasive intervention.	1C; strong agreement
8	CT: thin collimation and slice thickness of 5 mm or less and 100–150 ml (3 ml/s) during pancreatic/portal venous phase are recommended. MRI: axial FS-T2 and FS-T1 before and after *iv* gadolinium are recommended.	1C; strong
D	Fluid therapy	
9	Ringer’s lactate is recommended.	1B; strong
10a	Goal-directed therapy with 5–10 ml/kg/h should be used initially.	1B; weak
10b	Response should be assessed by either (1) heart rate (< 120/min), MAP (65–85 mmHg), and urinary output (> 0.5–1 ml/kg/h); (2) invasive clinical targets of stroke volume variation and intrathoracic blood volume; (3) hematocrit 35–44%.	2B; weak *t*
E	Intensive care management	
11	Patients fulfilling one or more parameters of the SCCM guidelines or with severe AP (according to Atlanta classification) should be treated in an IC setting.	1C; strong
12	Severe AP and AP requiring surgical/radiological or endoscopic intervention should be treated in a specialist center.	1C; strong agreement
13	Specialist centers are defined by high-volume, up-to-date IC facilities with the option for organ replacement therapy, daily access to interventional radiology/interventional endoscopy, and surgical expertise with necrotizing AP. Enrollment in prospective audits and into clinical trial whenever possible.	2C; weak *t*
14	Early fluid resuscitation (< 24 h) is associated with decreased rates of persistent SIRS and organ failure.	1C; strong agreement
15	Abdominal compartment syndrome (ACS) is defined as intraabdominal pressure > 20 mmHg with new-onset organ failure.	2B; strong
16	Medical treatment for ACS targets (1) hollow viscera volume, (2) intra-/extra-vascular fluid; (3) abdominal wall expansion. Invasive treatment options (when > 25 mmHg and persistent organ failure, multidisciplinary consent) include percutaneous drainage of ascites and surgical decompression. Retroperitoneum and omental bursa should be left intact.	2C; strong
F	Preventing infectious complications	
17	No routine antibiotic prophylaxis.	1B; strong
18	Selective gut decontamination might be helpful, but further studies are needed.	2B; weak
19	Probiotic prophylaxis is not recommended.	1B; strong
G	Nutritional support	
20	Oral feeding in predicted mild AP can be restarted once pain and inflammatory markers are decreasing.	2B; strong
21	Enteral tube feeding as the primary therapy in predicted severe AP.	1B; strong
22	Elemental or polymeric enteral nutrition can be used.	2B; strong
23	Nasojejunal or nasogastric route can be used for enteral nutrition.	2A; strong agreement
24	Parenteral nutrition as second-line therapy when nasojejunal tube is not tolerated and nutritional support is required.	2C; strong
H	Biliary tract management	
25	ERCP not indicated in mild biliary AP without cholangitis (1A) and probably not indicated in severe biliary AP without cholangitis (1B). ERCP probably indicated in biliary AP with common bile duct obstruction (1C). ERCP indicated in biliary AP with cholangitis (1B).	Evidence—see text; strong
26	Urgent ERCP (< 24 h) in patients with acute cholangitis. Evidence regarding optimal timing for ERCP in biliary AP without cholangitis is lacking	2C; strong
27	MRCP and EUS might prevent ERCPs for suspected common bile duct stones. EUS is superior to MRCP in detecting gallstones < 5 mm. MRCP is less invasive and more available. Neither technique clearly superior.	2C; strong
I	Indications for intervention in necrotizing AP	
28	Indications include (1) infected necrosis (suspected or documented) with clinical deterioration, preferably when walled-off; (2) ongoing organ failure for several weeks, preferably when necrosis is walled-off.	1C; strong
29	Routine percutaneous FNA to detect bacteria not indicated.	1C; strong a
30	Indications for intervention in sterile necrotizing AP: (1) ongoing gastric outlet obstruction/ biliary obstruction; (2) persistent symptoms; (3) disconnected duct syndrome. Necrosis should be walled-off.	2C; strong
J	Timing of intervention in necrotizing pancreatitis	
31	For infected necrosis, invasive interventions should be delayed until at least 4 weeks after initial presentation to allow walling-off.	1C; strong
32	Surgical necrosectomy should be delayed (for at least 4 weeks, until walled-off) regardless of patient subgroups.	
K	Intervention strategies in necrotizing AP	
33	Optimal strategy for infected necrotizing AP is either image guided percutaneous-retroperitoneal or endoscopic drainage, followed, if necessary, by endoscopic or surgical necrosectomy.	1A; strong
34	Percutaneous-retroperitoneal or endoscopic drainage should be the first step in the treatment of infected, walled-off AP.	1A; strong
35	Insufficient data to define subgroups of patients who would benefit from different treatments.	2C; strong agreement
L	Timing of cholecystectomy	
36	Mild biliary AP: cholecystectomy during index admission is recommended. Interval cholecystectomy is associated with recurrence.	1C; strong
37	Patients with peripancreatic collections: cholecystectomy should be delayed until collections resolve (or performed after 6 weeks if persisting collections are present).	2C; strong
38	Cholecystectomy recommended in patients after sphincterotomy for biliary AP.	2B; strong

Rapid diagnosis and prediction of severity are critical to provide fluid resuscitation and oxygen supplementation and intensive care for patients in need. The diagnosis requires at least two of the following three criteria: acute onset upper abdominal pain, serum amylase or lipase > 3x upper limit of normal, and/or imaging with contrast-enhanced CT or MRI. The revised Atlanta classification recommended the Modified Marshall scoring system for organ dysfunction that incorporates the renal, respiratory, and cardiovascular status of the patient, is easy to assess, and can be repeated daily [21]. Although the revised Atlanta classification recommended a systemic inflammatory response syndrome (SIRS) score of > 2 for severity prediction, this is no better than the clinically pragmatic and robust modified Glasgow system and serum CRP levels [12, 22, 23].

### Treatment strategies

Resuscitation with intravenous fluids, supplemental oxygen and close monitoring are essential from the outset of the attack. In gallstone AP, endoscopic retrograde cholangio-pancreatography (ERCP) and consecutive gallstone removal and sphincterotomy are indicated in patients with cholangitis or signs of persistent obstruction from choledocholithiasis [20, 24–27].

Predicted severe cases and/or those showing clinical deterioration with multi-organ dysfunction require management in the intensive care unit.

Both peripancreatic and pancreatic necrosis usually require treatment when infected, whereas sterile, non-obstructing necrosis can often be managed with a watch-and-wait strategy [7, 12, 20]. It is usual to delay intervention until the necrosis has walled-off, which usually takes approximately 4 weeks from symptom onset, but should not be delayed in the face of a deteriorating clinical scenario. Antibiotic treatment is indicated but only if infection of a necrotic collection is confirmed by fine needle aspiration or clinically suspected, and should be broad-spectrum and able to penetrate into the necrosis.

Left flank retroperitoneal pancreatic necrosectomy, percutaneous catheter and/or endoscopic transgastric drainage, laparoscopic approaches, minimally invasive retroperitoneal pancreatic necrosectomy (MARPN), and the step-up approach of percutaneous drainage with video-assisted open debridement have all been proposed as alternatives to open pancreatic necrosectomy [13–15, 28–38].

Debridement of infected pancreatic necrosis is the mainstay of treatment when percutaneous drainage fails, which is the case in 25 to 75% of patients. Open necrosectomy is associated with 34–95% morbidity and 6–25% mortality depending on the cohort, expertise, and the disease severity [14, 15, 17, 35, 36, 39–41]. In a collected retrospective multicenter study, high-risk cases of infected pancreatic necrosis had a mortality of 53% using open necrosectomy while less invasive endoscopic methods had a mortality of 38% [15, 29, 39–41].

### Surgical concepts: management of necrosis

Historically, surgery for necrosis in AP was associated with a very high mortality of up to 50% or higher [39, 42]. The timing of an intervention is important, with a general rule of thumb suggesting 4 weeks from the start of symptoms to enable walling-off of necrotic tissue, although this may not always be possible because of a deteriorating clinical scenario [20, 40, 43]. Other crucially important factors for the improvement of morbidity and mortality in the treatment of AP necrosis are advances and paradigm shifts in surgical technique.

#### Open pancreatic necrosectomy

The basic principle is the exposure of the necrotic area, usually after transection of the gastro-colic and duodeno-colic ligament, and blunt dissection then debridement of necrotic tissue. Sometimes, it is easier to enter the necrotic cavity as the necrosis invades the transverse colon in the space of Riolan, adjacent to the ligament of Treitz. Subsequently, the necrotic cavity can be managed in various ways as follows [40]:

1. Open Packing: the cavity is packed, and the patient is scheduled for repeat procedures, usually every 48 h, until the necrotic process is resolved; mortality of 12–49% with infected necrosis in 84–100% [40, 44].
2. Planned re-laparotomies: after initial necrosectomy and lavage, the patient is scheduled for re-laparotomy; mortality of 17–25% with infected necrosis in 75–79% [40, 45].
3. Closed continuous lavage: two or more large Salem-sump tubes are placed in the necrotic area and the ligaments are re-approximated to create a compartment, followed by high-volume continuous lavage immediately from the end of surgery; mortality of 12–49% with infected necrosis in 39–100% [40, 46, 47].
4. Closed packing: packing of the area is combined with insertion of Penrose drains and closed-suction drains; mortality of 6% reported in one series from the Massachusetts General Hospital in Boston of 64 patients with infected necrosis in only 56% [48].

Closed continuous lavage and closed packing are naturally associated with fewer re-interventions (no scheduled re-laparotomy) and fewer complications such as gastrointestinal or colonic fistulas and incisional hernias [40, 46–48]. In a subsequent series from the Massachusetts General Hospital of 167 patients, the mortality was 20.3% in patients receiving open debridement and closed packing in the first 28 days after symptom onset but only 5.1% with debridement and closed packing after 28 days [49]. A retrospective study from Finland of open necrosectomy in 109 had a 90-day mortality of 22.9% but with the subgroup being operated on after 28 days (*N* = 91), this was only 10.6% [50]. Longitudinal data from the Liverpool Pancreas Unit demonstrates a significant improvement in terms of mortality and overall complications for open pancreatic necrosectomy and minimally invasive approaches when comparing single-center data from 1997–2008 to 2008–2013 [14, 15].

In summary, open pancreatic necrosectomy can be a viable treatment option in selected patients. It remains the method of choice when other, less invasive options fail. However, other approaches have gained widespread acceptance over the last two decades and retroperitoneal access procedures represent the new standard of care that is going to be discussed below.

#### Minimal access retroperitoneal pancreatic necrosectomy

The advent of high-resolution contrast-enhanced computed tomography (CT) and sophisticated optical and surgical instrumentation enabled clinicians of the late twentieth century to assess different minimally invasive routes to pancreatic and peripancreatic necroses in AP patients [51–53].

Retroperitoneoscopic access emerged as one of the most promising techniques. Intraabdominal dissemination of infectious necrotic tissue and subsequent septic shock are avoided/minimized utilizing this extraperitoneal approach. Pioneered in Glasgow and Liverpool MARPN or minimal access retroperitoneal pancreatic necrosectomy (sometimes also referred to as minimally invasive retroperitoneal pancreatic necrosectomy = MIRPN) has influenced the development on minimally invasive approaches in centers across the world [14, 15, 29, 30]. MARPN is also referred to as “skunking” as the necrotic material being removed initially usually has a strong offensive odor.

##### Technique of minimal access retroperitoneal pancreatic necrosectomy (Fig. 2)

First, a 12-French catheter over a flexible guidewire is placed into the necrotic area under local anesthetic by a specialist interventional radiologist under CT guidance using a standard Seldinger technique. This follows a left flank line of retroperitoneal access between the spleen, the splenic flexure of the colon, and the left kidney [30]. The approach from the right flank is usually not possible because the duodenum and the inferior vena cava block the line of access into the head of the pancreas. The patient may then be transferred back to the intensive care unit or regular ward to await theater or go straight to the operating room. The patient is placed in a supine position with the left (or right) flank being elevated with a sandbag for better access. The patient may be given a light anesthetic, or alternatively the whole procedure can be conducted without any sedation or analgesia except for local anesthetic around the guidewire skin entry point to enable an incision in order to widen the track access site. The index case was an elderly patient with aortic stenosis and cardiovascular disease, undertaken only with local skin anesthetic who watched the whole procedure on the nephroscope video monitor.

**Fig. 2:**
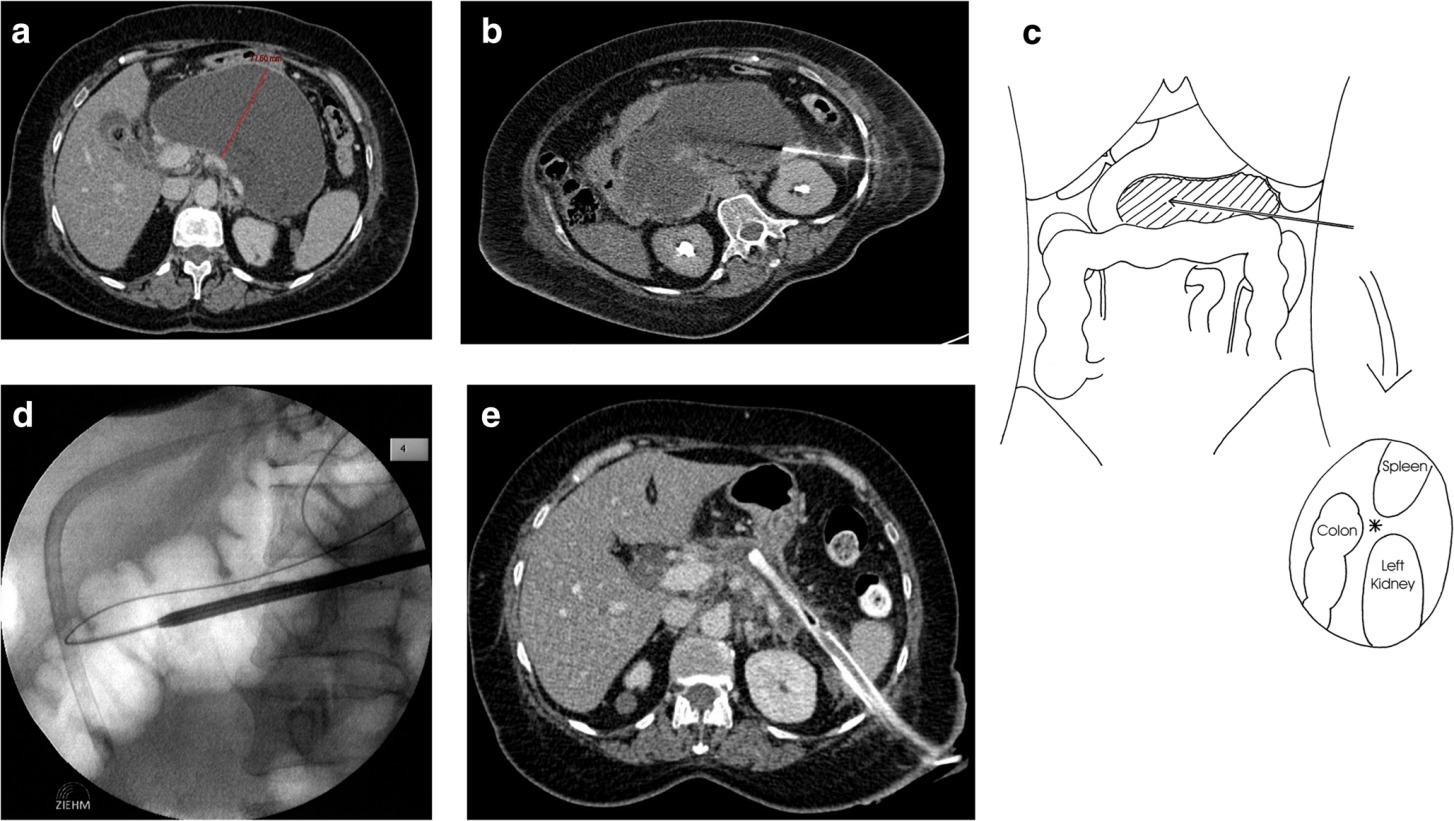
The serial treatment of necrotizing pancreatitis by MARPN (**a**). In the radiology department, the guidewire is inserted into the center of the necrotic mass, taking a line between the lower pole of the spleen, the splenic flexure of the colon, and the upper pole of the left kidney (**b**, **c**). Then in the operating room, the surgeon dilates the guidewire track using increasing diameter nephrectomy dilators, under X-ray control using the vertebral column and the position of a nasogastric tube as a reference point (**d**). Following multiple skunk procedures using a straight rigid nephroscope, the necrosis has largely been cleared and the necrotic cavity has collapsed around the 28-French chest drain (**e**); the tract will heal by granulation tissue on steady withdrawal then downsizing of the drain over several weeks as an outpatient

The catheter is then removed leaving the guidewire in situ under radiologic control by a C-arm. After confirmation of correct guidewire placement and incision of the skin, it is replaced by a plastic sheath, and the tract is serially dilated using a renal dilatation set. Once a 30-French tract has been established, a plastic sheath is left in place to prevent collapsing, and a rigid nephroscope with a video screen connection is used to visualize the necrosis. Continuous irrigation with saline-solution is essential to facilitate visualization of the cavity. Biopsy forceps are used to remove the black and/or gray necrotic tissue, which is readily distinguished from normal tissue which is pink or white.

An irrigation drainage system for continuous lavage on the ward is positioned under radiologic control. A bespoke drainage system is used comprising a pediatric 12-French nasogastric tube transfixed to the proximal tip of a large 28-French semi-rigid chest drain with additional holes cut out proximally and both tubes then suture-transfixed to the skin. The chest drain is connected to a large collecting bag (such as a urine bag) that can measure the fluid output and be emptied through a separate outlet tube. The rate of lavage should start at 3 L/24 h using normal saline and be commenced in the operating room to ensure unobstructed in-flow of saline and a steady outflow of lavage effluent. The inflow and outflow should be regularly recorded to ensure that the volumes are more or less equal over a 24-h period.

The main objective of the first procedure is to decompress the necrotic cavity which is normally under tension and send the pus and other infected tissues for culture and antibiotic sensitivities.

Several procedures are usually needed for a successful debridement and at the initial procedure, the surgeon should not aim to remove every necrotic piece that is visualized. For subsequent procedures, the plastic sheath and the nephroscope are reintroduced under visual control and no radiologic imaging is necessary except for the placement of the lavage drainage system. Again, these repeat procedures may not require a general anesthetic. The necrotic cavity will gradually collapse, and the necrotic areas will be replaced by healthy pink granulation tissue. Major vessels including the splenic and superior mesenteric arteries and superior mesenteric vein and the inferior vena cava may be seen. The steady collapse of the cavity can be radiologically monitored using soluble contrast injected into the cavity via the lavage system (a “tubogram”) and also by contrast-enhanced CT. As the patient steadily improves, the rate of lavage can be reduced but may need to be increased again if there is clinical deterioration. The progress of the patient can also be followed using sequential serum levels of CRP. Rising levels of CRP indicate the need for repeat skunking and/or new collections developing. Once the CRP falls to around 50 mg/L, further interventions are unlikely and when 30 mg/L or less the lavage may be stopped. At this stage, the chest drain tube is cut and a colostomy bag is placed around this to collect the effluent.

Most patients will be discharged home with a drainage bag in situ and followed up weekly. If the drain becomes blocked with thick debris and/or pus, it can be flushed with a small volume of normal saline. The drainage tube is shortened by 2–5 cm every week or so to enable the sinus tract to granulate towards the skin. With 10–15 cm remaining, the chest drain is downsized to a tube with a smaller diameter such as an 18-French nasogastric tube cut to size. It is important to allow the tract to fully granulate before the skin closes over the tract; otherwise, this will cause a subcutaneous abscess.

MARPN was initially limited to necroses in the pancreatic tail and body. Over time, indications have been expanded to necroses in the paracolic gutters usually by additional access tracts aiming caudally from the left and/or right flanks. Additional drainage tubes may be required for pelvic collections and transgastric or transduodenal routes for endoscopic drainage of the head of the pancreas if left-sided access is insufficient for complete debridement. A single multipurpose laparoscopic port inserted extra-peritoneally may facilitate access to paracolic necrotic collections, using a zero-degree nephroscope, and an articulated grasper and continuous irrigation/drainage, but of course not entering the peritoneal cavity. These combined procedures are classified as complex MARPNs [15]. This means that, in patients where debridement is indicated, the majority of cases can be treated via retroperitoneoscopic approaches without entering the peritoneal cavity and avoiding large abdominal incisions.

##### Results of minimal access retroperitoneal pancreatic necrosectomy

In the original series from Glasgow, there were two (14.3%) deaths from 14 patients with infected pancreatic necrosis treated by MARPN [29]. In a small series of 18 patients with infected pancreatic necrosis from Beijing, all were successfully treated by MARPN without morbidity or mortality [54]. In the series of 394 patients with pancreatic necrosis (77.7% infected) from Liverpool, the mortality was lower in MARPN-treated patients (15.3–19%) compared with that treated with open pancreatic necrosectomy (23.3–38%) [14, 25]. The rate of post-operative multi-organ failure is lower after MARPN (20.4–31% versus 35–56% respectively), and there are fewer post-operative complications (55.0–63.5% versus 81.0–81.7%) [14, 15].

#### Left flank necrosectomy and video-assisted retroperitoneal debridement

In 1989, Fagniez and colleagues from Créteil, France, described a direct retroperitoneal approach for necrosectomy in 40 patients with severe acute pancreatitis, all but one with infected necrosis, and 18 had failed pancreatic surgery elsewhere [28]. The approach was with a left lateral incision, just anterior to the 12th rib, allowing direct access to the pancreas and a complete manual exploration of the gland and peripancreatic spaces. Thirteen patients (32.5%) died, although only four patients (18.2%) out of the 22 operated on primarily in Créteil. Twenty patients (50%) developed a local complication including major hemorrhage in eight and eleven with colonic fistula and/or necrosis, and one with a gastric fistula. Respiratory failure developed in ten patients and another seven patients developed multi-organ failure [28].

Van Santvoort et al. adopted video-assisted retroperitoneal debridement (VARD) beginning with a left flank sub-costal incision to directly remove necrosis followed by a laparoscope to access deeper lying necrosis and then using continuous lavage [55]. They described as “a hybrid between pure endoscopic retroperitoneal necrosectomy and the open (20 cm incision) translumbar approach, described by Fagniez et al. in 1989” [55]. Unfortunately, it is not possible to obtain any clear outcome data on the VARD procedure as it is contained within the “step-up approach” that provides combined outcome data with prior percutaneous drainage [31, 34].

In the PANTER multi-center trial from the Netherlands, 88 patients with necrotizing pancreatitis were randomly assigned to undergo up-front open necrosectomy with continuous lavage or a step-up approach combining initial percutaneous drainage and if there was no clinical improvement, then necrosectomy by VARD [31]. The primary endpoint was a composite of major complications which included new-onset multiple organ failure or multiple systemic complications, perforation of a visceral organ or enterocutaneous fistula, or bleeding, or death. The primary endpoint occurred in 31 (69%) of 45 patients assigned to open necrosectomy and in 17 (40%) of 43 patients assigned to the step-up approach. In the step-up approach group, 17 patients had percutaneous drainage only but with two (11.8%) deaths. There were six (23.1%) deaths in the remaining 26 patients who had VARD, compared with seven (15.6%) deaths in the 45 patients that had open necrosectomy [31]. Follow-up showed that in the step-up group, patients had fewer incisional hernias and less exocrine insufficiency (not surprisingly but there were no differences between the groups in terms of recurrent acute or chronic pancreatitis, endoscopic or surgical interventions pancreatic, quality of life, or costs) [36].

#### Endoscopic transgastric necrosectomy

The Dutch Pancreatitis Study Group subsequently undertook a multicenter study in which patients were randomly assigned to one of two step-up groups, the endoscopic approach (*N* = 51) consisting of endoscopic ultrasound-guided transluminal drainage followed, if necessary, by endoscopic necrosectomy and the surgical approach consisting of percutaneous catheter drainage followed, if necessary, by VARD (*N* = 47) [34]. Endoscopic necrosectomy was undertaken in 29 (56.8%) patients randomized to the endoscopy group and VARD was undertaken in 23 (51.1%) patients in the surgery group [34]. There were nine (18%) deaths in the endoscopy group and six (13%) patients in the surgery group but we do not know how many deaths occurred before the endoscopic intervention or VARD; major complications or death occurred in 22 (43%) and 21 (45%) patients, respectively [34].

A single-center study from Florida Hospital in Orlando, USA, randomized 32 of 66 patients with pancreatic necrosis to minimally invasive surgery, either laparoscopic cysto-gastrostomy or VARD depending on location of collection, and 34 patients to an endoscopic step-up approach comprising transluminal drainage with or without necrosectomy. The primary endpoint was a composite of major complications of new-onset multiple organ failure, new-onset systemic dysfunction, enteral or pancreatic-cutaneous fistula, bleeding, and perforation of a visceral organ or death during 6 months of follow-up. Death occurred in two (6.3%) patients in the surgery group and in three (8.8%) patients in the endoscopy group; the primary endpoint occurred in four (11.8%) patients who received endoscopic procedure and in 13 (40.6%) patients who had minimally invasive surgery [56].

#### Comparison of outcomes of different techniques

Comparison of the different techniques is not straightforward as:

1. Different techniques are used sequentially on the same patient thereby altering the characteristics of the outcomes for each type of technique, thus introducing a selection bias [57];
2. Description of key characteristic features of the pancreatitis may not be included such as the presence or absence of pancreatic necrosis, extent of pancreatic necrosis, transient or persistent organ failure, and pre-operative and post-operative intensive therapy unit (ITU) requirement;
3. Details of infected or sterile pancreatic necrosis before intervention are missing, as once intervention takes place, infection is almost invariably acquired, so falsely reported as being infected necrosis;
4. Selective bias with late referrals of patients: for example, in the three-center (Calgary, Stanford, and Indiana) retrospective study on surgical transgastric necrosectomy for necrotizing pancreatitis as a single-stage procedure for walled-off pancreatic necrosis, the median delay from symptom onset to surgical treatment was 53, 60, and 71 days, respectively, leaving only the relatively low-risk survivors to have the surgery [37];
5. Lack of a clear distinction between acute peripancreatic fluid collections (which do not need treatment), pancreatic pseudocysts (which usually resolve without treatment), pancreatic necrosis, acute necrotic collection, and walled of necrosis, all with different outcomes;
6. Grouping various minimized techniques together, such as MARPN, VARD, and endoscopic transgastric necrosectomy (ETN), when they are probably not comparable in terms of outcomes [35, 58];
7. Use of composite endpoints where significant differences might be seen due to potentially biased observations such as pancreatic enzyme replacement therapy, or pancreatic fistula (which by definition always occurs with a percutaneous approach), while unbiased events such as death (when there may be no significant difference) assume a secondary significance [56].

Considering these reservations, data for various techniques are shown in Table 4 for comparison. Although VARD has been promoted, the data on how effective it is are rather small compared with MARPN [15, 31]. A recent series of 179 consecutive patients with necrotizing pancreatitis from the Massachusetts General Hospital, Boston, USA, revealed a 90-day mortality rate of only 2/91 (2.2%) in patients treated by a variety of minimally invasive techniques (including ETN, STE, and VARD) compared with 9/88 (10.2%) in patients treated by open necrosectomy [58]. The International Association of Pancreatology/American Pancreatic Association guidelines recommend either a conventional or endoscopic step-up approach as the initial treatment strategy of choice in patients with infected necrosis or persistent organ failure and necrosis [20]. One advantage of this approach is that more invasive interventions of incorrectly diagnosed necrotic collections—that are actually acute peripancreatic fluid collections (that do not need treatment) or pseudocysts (that mostly resolve)—are avoided. One disadvantage is that initial drainage only leads to partial resolution of a necrotic collection that subsequently complicates minimal access approaches, forcing an open necrosectomy that would have been otherwise avoidable.

**Table 4:** Comparison of various techniques for treating pancreatic necrosectomy

Technique reference	Patient number	Death number	Infected necrosis number*	Pre-op. ITU number	Post-op. ITU number	Comment
Percutaneous drainage						
Drainage only. Van Santvoort HC et al. (2010).	17	2 (11.8%)				PANTER multicenter Dutch trial *n* = 88 patients with randomized to open necrosectomy with continuous lavage or a step-up approach of percutaneous drainage and if no clinical improvement then VARD.
Drainage as first intervention. Drainage only. Drainage then necrosectomy: laparotomy = 25; VARD = 44; ETN = 7. Van Santvoort HC, et al. (2011)	130 54 76	26 (20%) 9 (16.7%) 17 (22.4%)	NA	NA	NA	From 639 consecutive patients 2004–2008, in 21 Dutch hospitals; pancreatic necrosis in 324 (51%); infected in 202 (31.6%). Percutaneous, *n* = 113; endoscopic transluminal (*n* = 17).
Open necrosectomy						
Open necrosectomy with closed continuous lavage. Beger HG et al. (1988)	95	8 (8.4%)	37/89 (42%)	NA	NA	Single-center 744 consecutive patients, Ulm Germany, 1982–1987; 567 with edematous pancreatitis (4 deaths, 0.7%).
Re-operated on demand = 196 (72.6%); planned re-laparotomies − 74 (27.4%); all drainage by open packing, laparostomy, or both. Götzinger P et al. (2002)	340	133 (39.1%)	154 (45.3%)	340 (100%)	340 (100%)	Prospective consecutive patients needing surgery from two hospitals in Vienna Austria for severe acute pancreatitis, all needing ITU. An additional 101 (29.7%) patients developed infected necrosis.
Open necrosectomy followed by closed packing and drainage. Total Infected Sterile Rodriguez JR et al. (2008)	167 113 45	19 (11.4%) 17 (15.0%) 2 (4.4%)	113 (67.7%) 113 0	NA NA	92 (55.5%) 72 (63.7%) 20 (44.4%)	Single-center series MGH, Boston, USA, 1990–2005 of 2449 consecutive patients with acute pancreatitis, 167 (6.8%) with surgical necrotizing pancreatitis.
Open necrosectomy with closed continuous lavage. Van Santvoort HC, et al. (2010)	45	7 (15.6%)	42 (93%)	21 (47%)	NA	PANTER multicenter Dutch trial (*n* = 88) randomized to open necrosectomy with continuous lavage (*n* = 45) or step-up using percutaneous drainage and if no clinical improvement then VARD (*n* = 43).
van Santvoort HC, et al. (2011)	68	48 (70.6%)	NA	NA	NA	From 639 consecutive patients 2004–2008, in 21 Dutch hospitals; pancreatic necrosis 324 (51%); infected necrosis 202 (31.6%).
Open necrosectomy with closed continuous lavage. Gomatos IP et al. (2016)	120	28 (23.3%)	60 (50%)	36 (30%)	90 (75%)	From consecutive 394 Patients, single-center series, Liverpool, 1996–2013 inclusive.
Van Brunschot S, Hollemans RA, et al. (2018)	376	87 (23.1%)	333(88.6%)	NA	NA	Retrospective data of 1167 patients from 51 hospitals in 15 cohorts; 198 patients derived after matching.
Open necrosectomy with drains but no lavage; reoperation on demand. Husu JL et al. (2020)	109	25 (22.9%)	85 (78.0%)	44 (44.4%)	NA	Retrospective single-center consecutive series, Meilahti Hospital, Helsinki Finland, 2006–2017; 52 (47.7%) patients had a reoperation; 27 (24.8%) had a re-necrosectomy < 6 months of the index operation.
Open necrosectomy followed by closed packing and drainage. Luckhurst CM et al. (2020)	88	9 (10.2%)	63 (71.6%)	NA	55 (62.5%)	Single-center series MGH, Boston, USA, 2006–2019 of 179 consecutive patients with necrotizing pancreatitis treated either by open necrosectomy (*n* = 88) or minimally invasive surgery (*n* = 91): ETN = 29; STE = 14; ETN + STE = 10; VARD = 7; other = 16.
Left flank necrosectomy						
Total. Primary procedure. Previously failed surgery. Fagniez PL et al. (1989)	40 22 18	13 (32.5%) 4 (18.2%) 9 (50%)	18 (45%) NA NA	40 (100%) 22 (100%) 18 (100%)	40 (100%) 22 (100%) 18 (100%)	Consecutive single-center series Créteil, France; 22 operated on primarily; 18 had failed pancreatic surgery elsewhere
Minimal access retroperitoneal pancreatic necrosectomy (MARPN)						
Carter R et al. (2000)	14	2 (14.3%)	14 (100%)	7(50%)	8 (57.1%)	14 consecutive patients, single-center series, Glasgow, UK.
Gomatos IP et al. (2016)	274	42 (15.3%)	162 (59.1%)	103 (37.6%)	112 (40.9%)	From consecutive 394 Patients, single-center series, Liverpool, UK, 1996–2013 inclusive. Mortality 2009–2013 inclusive = 13 of 124 (10.5%) patients.
Wang PF et al. (2018)	18	0 (0%)	18 (100%)	NA	NA	Single-center series, Beijing, China, during 2017: 9 patients had moderately severe acute pancreatitis, and the other 9 patients had severe acute pancreatitis.
Video-assisted retroperitoneal debridement (VARD)						
VARD following catheter drainage. Van Santvoort HC, et al. (2010).	26	6 (23.1%)	NA	NA	NA	PANTER multicenter Dutch trial (*n* = 88) randomized to open necrosectomy (*n* = 45) with continuous lavage or a step-up using percutaneous drainage and if no clinical improvement then VARD (*n* = 43).
Endoscopic transgastric necrosectomy (ETN)						
Van Brunschot S, Hollemans RA, et al. (2018)	198	17 (8.6%)	135 (68.2%)	NA	NA	Retrospective data of 1167 patients from 51 hospitals in 15 cohorts; 198 patients derived after matching.

*ITU*, intensive therapy unit; *VARD*, video-assisted retroperitoneal debridement; *ETN*, endoscopic transgastric necrosectomy; *STE*, sinus tract endoscopy; *MARPN*, minimal access retroperitoneal pancreatic necrosectomy

*Infected pancreatic necrosis diagnosed prior to intervention or during the first intervention

The American Gastroenterological Association (AGA) Clinical Practice Update on the management of pancreatic necrosis recommends that the use of direct endoscopic necrosectomy should be reserved for those patients with limited necrosis who do not adequately respond to endoscopic transmural drainage using large-bore, self-expanding metal stents/lumen-apposing metal stents alone or plastic stents combined with irrigation [59]. The AGA also recommends that minimally invasive operative approaches to the debridement of acute necrotizing pancreatitis are to be preferred to open surgical necrosectomy whenever possible [59].

### Other complications and their surgical management

#### Colonic and enteric fistula

Enteric and especially colonic necrosis, ischemia, and hemorrhage in the context of severe AP are usually caused by the spread of pancreatic enzymes and pancreatic/peripancreatic necrosis. If suspected, colonic resection is essential [15, 60]. Colonic fistulas, which can appear in 17–19% of patients, are associated with increased mortality [61]. This complication can be managed without surgery utilizing percutaneous drainage in around 47% of cases. In a large series including 132 patients with colonic fistula, mortality in patients requiring surgical intervention for colonic fistula was higher (37%) compared with the group receiving percutaneous drainage (19%) [61].

#### Hemorrhage

Pancreatic fistula and necrosis can erode blood vessels involved in the collection causing major bleeding and occurs in 15–18% of cases and pseudo-aneurysm in around 4% [15]. With open pancreatic necrosectomy, the mortality is very high. In a collected series of 44 cases reported in 2003, the overall mortality rate was 34.1% [62]. The splenic artery, portal vein, spleen, and unspecified peripancreatic vessels were the most commonly involved sources of bleeding, with associated mortality rates of 33.3%, 50.0%, 30%, and 28.5%, respectively [62]. Massive hemorrhage was more frequently associated with severe necrosis, with a mortality rate of 37.9% [62]. Earlier on in the Liverpool series, the mortality rate was 70% when attempted treatment for massive hemorrhage was straight to open laparotomy and packing [63]. Subsequently, for bleeding in patients who had MARPN, tamponade of the bleeding was introduced simply by stopping the continuous irrigation and clamping the chest drain with arterial forceps; in most cases, the bleeding would be controlled without the need for further intervention. For open necrosectomy, the standard procedure for bleeding is emergency angiography and embolization, and only if this is not successful should there be a laparotomy followed by packing to control the bleeding. Using this approach, the mortality was only 16% from bleeding in the Liverpool series [14].

#### Disconnected main pancreatic duct

Disconnected main pancreatic duct (DPD) is defined as a discontinuity of the main pancreatic duct (MPD) and is a feature of severe necrotizing pancreatitis with central pancreatic necrosis first described in 1993 [64]. An external pancreatic fistula will therefore be usually expected using MARPN/VARD/percutaneous drainage for severe necrotizing pancreatitis. It can be managed as an outpatient procedure by gradual shortening of external drain then downsizing using a smaller French diameter nasogastric tube cut to size and aggressive endoscopic or surgical interventions are not usually necessary [65]. Late recurrence leading to a drain tract pseudocyst can occasionally occur due to a distal structure in the neck or head of the pancreas and is then best managed by Roux-en-Y pseudocyst-enterostomy [65].

#### Abdominal compartment syndrome

Severe AP is accompanied by drastic compartment fluid shifts especially into the interstitial spaces leading to abdominal compartment syndrome. Although laparostomy has been suggested as a means of treatment, there is no quality evidence to support this and may well be deleterious to the condition of the patient.

### Timing of cholecystectomy

Cholecystectomy is recommended for patients with gallstone associated pancreatitis in order  to prevent further attacks and should be undertaken at the index admission is recommended for mild biliary pancreatitis [20, 66]. For patients with peripancreatic collections, however, cholecystectomy should be delayed until the collections have resolved or until after 6 weeks as there is a higher risk for sepsis [20, 67].

### Summary and outlook

Severe AP still is a life-threatening condition requiring a multidisciplinary approach. An accurate diagnosis should be made a soon as possible, and initiating resuscitation with large volume intravenous fluids and oxygen by mask. If there is any doubt of the diagnosis, then an urgent contrast-enhanced CT scan should be undertaken. If severe disease is predicted using clinical assessment and serum CRP > 150 mg/L, the patient will require intensive monitoring. Most deaths within the first week or so are due to multi-organ failure so severe cases will require management on the intensive therapy unit. During the second phase of the disease, death is due to local complications arising from the pancreatic inflammation. Accurate identification of these local complications is required to determine the correct form of treatment. Acute peripancreatic fluid collections are common, not requiring any treatment. Most pancreatic pseudocysts also largely resolve on conservative management, not needing intervention. Necrotizing pancreatitis causing acute necrotic collections and later walled-off necrosis will require treatment if symptomatic or infected. Initial endoscopic transgastric or percutaneous drainage will resolve less serious collections but necrosectomy using minimally invasive approaches will be needed for more serious collections and usually require a combination of techniques for larger extensive collections. To prevent recurrent attacks of AP, then causative factors need to be removed where possible. Future progress needs to be focused on better management of multi-organ failure in the first phase and more effective minimally invasive techniques for removal of necrosis.
